# Myeloid-Derived Suppressor Cells Are Regulated by Estradiol and Are a Predictive Marker for IVF Outcome

**DOI:** 10.3389/fendo.2019.00521

**Published:** 2019-07-30

**Authors:** Cong Hu, Yu Zhen, Bo Pang, Xiuying Lin, Huanfa Yi

**Affiliations:** ^1^Central Laboratory of the Eastern Division, The First Hospital of Jilin University, Changchun, China; ^2^Center for Reproductive Medicine, Center for Prenatal Diagnosis, The First Hospital of Jilin University, Changchun, China; ^3^Department of Dermatology, The First Hospital of Jilin University, Changchun, China; ^4^Department of Cardiology, The First Hospital of Jilin University, Changchun, China; ^5^Center for Reproductive Medicine, Jilin Province People's Hospital, Changchun, China

**Keywords:** estradiol, VEGF, MDSC, protein–protein interactions, IVF

## Abstract

*In vitro* fertilization (IVF) is an effective means to treat infertility, but the pregnancy rate is still unsatisfactory and reliable markers to predict pregnancy outcome are ill-defined. Myeloid-derived suppressor cell (MDSC) are critically involved in decisions related to the acceptance or rejection of foreign fetal antigens by the maternal immune system. However, factors that regulate peripheral blood MDSC during pre-pregnancy are poorly defined. Thus, the goal of this study was to assess the relationships among serum estradiol (E_2_) and endothelial growth factor (VEGF) levels, MDSC ratios, and pregnancy outcome associated with IVF. Patients undergoing IVF treatment (*n* = 54) were recruited from January to June 2018. Levels of E_2_ and VEGF were measured by ELISA, MDSC ratios among peripheral blood mononuclear cells (PBMC) were detected by flow cytometry, and the crosstalk among these parameters was analyzed. A receiver operating characteristic curve (ROC) of MDSC levels was plotted to assess this measure as an independent predictive factor for pregnancy. In addition, we analyzed the possible involvement of molecular pathways by bioinformatics. When E_2_ levels were <4,000 pg/ml, MDSC proportion was positively correlated with serum E_2_ and VEGF levels. However, when E_2_ levels were >4,000 pg/ml, MDSC ratio and VEGF levels were negatively correlated with E_2_. A ROC curve revealed that the percentage of MDSC had better sensitivity and specificity at a concentration of 8.22% (0.875 and 0.75, respectively; area under the curve (AUC) = 0.859) to predict pregnancy success, based on multiple logistic regression analysis. Furthermore, we found 12 target genes of E_2_ and VEGF, and also functional genes related to MDSC, indicating potential protein–protein interactions underlying these associations. In summary, we showed that E_2_, depending on its concentration, might play a dichotomous role in influencing the MDSC proportion by regulating VEGF. In IVF patients, an increased MDSC ratio among PBMC was highly correlated with elevated pregnancy rates, independent of the effects of E_2_, which might provide new insight into immune-related miscarriage and IVF failure.

## Introduction

Successful pregnancy is a concerted process, involving multiple mechanisms to prevent rejection of the semi-allograft fetus, which is immunologically foreign to the mother. Although *in vitro* fertilization (IVF) is an effective means to treat infertility, the pregnancy rate is still unsatisfactory. Upon embryo implantation, the mother's immune system develops an immunological tolerance response to prevent miscarriage (1, 2). Dysfunctions in the maternal immune system can lead to miscarriages or severe complications during pregnancy such as preeclampsia (3) and preterm deliveries (4). Moreover, estrogen was shown to augment the secretion of inflammatory cytokines such as TNF-α, IL-6, and IFNγ in Th1 lymphocytes (5), and can also stimulate the expression of key Th17 cell transcription factors by activating STAT3 and the expression of RORα and RORγ (6). Supraphysiologic estrogen during IVF makes the body inclined to generate pro-inflammatory responses, to stabilize the immune-associated microenvironment, some anti-inflammatory immune cells can be recruited. Although some subsets of immune cells participate in maternal–fetal immune tolerance, the underlying mechanisms are still not clear (7). We postulated that E_2_ might affect immune tolerance during controlled ovarian stimulation (COS). Understanding such mechanisms will facilitate the development of effective therapies to improve IVF outcome.

Myeloid-derived suppressor cell (MDSC), which plays a pivotal role in suppressing innate and adaptive immune responses in the tumor microenvironment (8, 9), are defined as heterogeneous myeloid precursor cells that expand in the blood, lymphatic organs, and tumor sites (10, 11). Human MDSC is classified into two subsets, namely granulocytic MDSC (G-MDSC; CD11b^+^ HLA-DR^low/−^CD33^+^CD15/CD66b^+^) and monocytic MDSC (M-MDSC, CD11b^+^ HLA-DR^low/−^CD33^+^CD14^+^) (12, 13). These cells induce and maintain immune tolerance by inhibiting T cell activation and proliferation, suppressing natural killer cell cytotoxicity, inducing Foxp3 expression in CD4^+^CD25^−^ T cells, and blocking macrophage-induced IL-12 (14). Several factors including hematopoietic and vascular growth factors, pro- and anti-inflammatory cytokines, and human leukocyte antigens induce the accumulation of MDSC to elicit and maintain maternal–fetal interface immune tolerance during pregnancy (14, 15). In addition to maintaining immune tolerance, MDSC can also promote angiogenesis during placental development and the remodeling of maternal uterine spiral arteries during pregnancy (15). Accordingly, recent studies showed that MDSC numbers were significantly elevated in pregnant women and decreased in the blood and endometrium during miscarriage (16, 17).

VEGF is a key regulator of angiogenesis and vascular function (18); it not only recruits MDSC to play an immunosuppressive role (19), but also inhibits the function of T cells (20). Further, MDSC can secrete VEGF, which plays an important role in reconstruction of the maternal uterine spiral artery and placental development during pregnancy (15). Although, E_2_ levels have been proposed to be the critical determinant of implantation (21), there is still lack of predictive marker for pregnancy outcome. In addition, factors that modulate peripheral blood MDSC during pre-pregnancy are poorly-defined. Thus, here, we investigated the relationships among serum E_2_ and VEGF levels, MDSC ratios, and pregnancy outcome in IVF patients and assessed possible associated molecular pathways by bioinformatics.

## Materials and Methods

### Patients

Women of an average age of 28.77 ± 3.75 years, undergoing IVF treatment (*n* = 54) were recruited from January to June 2018 at the Reproductive Medicine Center of Jilin Province People's Hospital. The inclusion criteria were as follows: (I) first IVF/Intracytoplasmic sperm injection (ICSI) cycle, (II) ≤35 years of age, (III) good ovarian reserve, (IV) normal hormones and karyotype, (V) without any known immunological diseases, (VI) without uterine abnormalities (fibroid, uterine septum, and uterine polyp), and (VII) without endometriosis. The exclusion criteria were as follows: (I) poor IVF/ICSI stimulation response (≤5 oocytes collected), (II) dissatisfactory appearance of endometrium at HCG day (thickness < 8 mm), (III) progesterone > 2.0 ng/ml at HCG day, (IV) poor embryo development (<2 high-quality embryos), and (V) miscarriage after pregnancy.

### *In vitro* Fertilization and Embryo Transfer

All patients were treated with the luteal-phase GnRH-a protocol. Ovarian stimulation was induced with recombinant follicle-stimulating hormone (FSH; Gonal-F, Merck Serono, Switzerland) and human menopausal gonadotropin (HMG; menotrophins for injection, Livzon, China) after pituitary suppression was achieved with GnRH-a (Triptorelin Acetate Injection, Tiantaishan Pharmaceuticals, China). Chorionic gonadotrophin for injection (HCG, Vidrel, Merck Serono, Switzerland) was administered when the dominant follicles (two or more) reached 18 mm in diameter. Oocytes were collected 36 h after HCG administration with vaginal ultrasound-guided follicular aspiration. Mature oocytes were incubated at 37°C with 5% CO_2_ and then inseminated with spermatozoa 4–6 h after oocyte retrieval for IVF or ICSI according to the quality of sperm. The oocyte retrieval day was defined as day 0. Then, the embryos were scored on day 3 on a scale of 1–5 according to Scott's scoring system (6). Embryos of grades 1 and 2 were defined as high-quality embryos. The low-quality embryos were cultured to blastocysts. All surplus embryos and blastocysts were vitrified.

Intrauterine administration was performed at the estrogen-primed frozen embryo transfer (FET) cycle. The patients were administered a daily dose of 4–6 mg of estradiol valerate (Progynova; Bayer Schering Pharma, France) from menstrual cycle day 2 to prepare the endometrium and ultrasonography was performed to measure the endometrium until 8 mm, after which, the estradiol dose was adjusted, and treatment lasted for at least 10 days. Luteal phase support was performed using 90 mg of vaginal progesterone (Crinone gel 8%; Merck, Germany), administered from 3 days before the FET day. Two high-quality embryos were transferred into an adequately prepared endometrium. Serum β-hCG levels were measured from peripheral blood 2 weeks after FET. Those with positive β-hCG results underwent ultrasonography another 2 weeks later to confirm clinical pregnancy. Clinical pregnancy was defined as the presence of an intrauterine gestational sac. Luteal phase support was continued until 9 weeks of pregnancy.

E_2_, VEGF, and MDSC assays were performed at a random day during ovarian stimulation, but before follicular maturation, and also on the day of FET. A sample of 2 ml of peripheral blood was also collected.

### Peripheral Blood Mononuclear Cell (PBMC) Isolation and Flow Cytometry

Peripheral blood was collected in sterile heparinized tubes from each patient, and PBMC, obtained by Ficoll–Hypaque gradient centrifugation, were analyzed by flow cytometry. The following anti-human fluorescence conjugated antibodies and their corresponding isotype controls used were as follows: anti-human CD11b-APC-cy7 (clone ICRF44, BD), anti-human HLA-DR-PE (clone G46-6, BD), anti-human CD14-FITC (clone 63D3, Biolegend), and anti-human CD66b-Percpcy5.5 (clone G10F5, Biolegend), FVD (BD), CD3-PE-cy7 (clone HIT3a, Biolegend), CD4-APC (clone OKT4, Biolegend), CD8-PB (clone RPA-T8, Biolegend). We used FVD to discriminate dead cells, and MDSC were classified into two subsets, G-MDSC (CD11b^+^ HLA-DR^low/−^ CD66b^+^) and M-MDSC (CD11b^+^ HLA-DR^low/^CD14^+^). Data were analyzed with FlowJo 10.0 software package (Treestar Inc., Ashland, OR, USA).

For flow cytometric sorting, an Aria II fluorescence activated cell sorter (BD, Mountain View, CA, USA) was used. The strategy for MDSC sorting was CD11b^+^ HLA-DR^low/−^ cells from live PBMC. Depletion of MDSC was performed by harvesting the remaining PBMC after MDSC sorting.

### Cells Culture

PBMC was collected from an infertile patient on menstrual cycle day 2, with basal hormone levels, and transferred to dishes with RPMI 1640 medium (Gibco, Carlsbad, USA) supplemented with 2 mM L-glutamine, 10 mM HEPES, 20 mM 2-methoxyestradiol, 150 U/ml streptomycin, 200 U/ml penicillin, and 10% fetal bovine serum. Cells (2 × 105) were cultured in 96-well plates and treated with various concentrations (0, 10, 20, 40, and 100 μM) of 17β-estradiol (Sigma-Aldrich, China). The cultures were maintained at 37°C in a 5% CO_2_-humidified atmosphere in 96-well plates. Medium was changed on the third day. The proportion of CD11b+HLA-DRlow/- MDSC was analyzed by flow cytometry on the sixth day.

### T Cell Proliferation Assay

T cell proliferation was evaluated by carboxyfluorescein succinimidyl ester (CFSE) dilution. PBMC were labeled with CFSE (2.5 μM) (Invitrogen, Carlsbad, CA, USA) and then stimulated with anti-CD3/CD28 antibodies (2 and 1 μg/ml, separately) (eBioscience). After culturing whole PBMC or those depleted of MDSC for 3 days, T cell proliferation was determined by a flow cytometer. No stimulation served as a negative control.

### ELISA

Serum samples collected from patients were kept at −80°C. Enzyme immunoassays were performed to determine the concentration of VEGF and S100A9 using ELISA kits (Beinuo Biotechnology, Shanghai, China and Biolegend, San Diego, CA, USA, separately) (*n* = 23), according to the manufacturer's instructions. All samples were assayed in duplicate.

### Real-Time Polymerase Chain Reaction Analysis

Initially, total RNA was extracted from PBMC using AxyPrep Multisource Total RNA Miniprep Kit (Axygen, Union City, CA, USA). The RNA quality was checked based on the A_260_/A_280_ ratio, and pure RNA samples were converted to cDNA using PrimeScript RT Reagent Kit with gDNA Eraser in accordance with the manufacturer's instructions (Takara Bio, Kusatsu, Japan). The polymerase chain reaction was performed using SYBR Green based on the instruction manual (Takara Bio, Kusatsu, Japan). The primer sequences are presented in Table 1 (Comate Bioscience, Changchun, China). The relative mRNA expression of different genes was quantified using the ΔΔCt method. β*-actin* was used as a housekeeping gene.

**Table 1:** Oligonucleotide and primer sequence.

**Oligonucleotide**	**Primer sequence**
β-actin	5′-TTCAACACCCCAGCCATG-3′ (forward), 5′-CCTCGTAGATGGGCACAGT-3′ (reverse)
S100A9	5′-GGTCATAGAACACATCATGGAGG-3′ (forward), 5′-GGCCTGGCTTATGGTGGTG-3′ (reverse)

### Hormone Assays

Serum E_2_ and progesterone (P) were measured by electro-chemiluminescence immunoassay (ECLIA, Cobas Roche Diagnostics, Mannheim, Germany). Intra- and inter-assay variations were <8 and 11%, respectively.

### Analysis by GeneMANIA

GeneMANIA is a flexible, user-friendly web interface used for generating hypotheses regarding gene functions, analyzing gene lists, and prioritizing genes for functional assays (22). After selecting *Homo sapiens* from the nine optional organisms, the genes of interest were entered into the search bar and the results were collated.

### GO Analysis, Pathway Analysis, and Network Construction

DAVID, a powerful tool for network biology, comprises an integrated biological knowledge base and analytic tools aimed at systematically extracting biological significance from large lists of genes or proteins (23). Potential targets were uploaded to the DAVID 6.8 server (https://david.ncifcrf.gov/home.jsp) for GO analysis, which was used to identify functionally-related genes from the list of differentially-expressed genes. Functionally related genes categorized based on biological processes, cellular components, and molecular functions were grouped by DAVID. A false discovery rate (FDR) < 5.0 and *p* < 0.05 were considered statistically significant. To achieve a systematic understanding of the inter-relationships among compounds, targets, and diseases, compound-target-pathway networks were constructed and analyzed in Cytoscape 3.3.

### Statistical Analysis

For the development, validation, and reporting of the proposed risk score, we followed the TRIPOD (Transparent Reporting of a Multivariable Prediction Model for Individual Prognosis or Diagnosis) guideline (24). GraphPad Prism version 7.00 and SPSS version 21.0 software were used for statistical analysis. All data were presented as the mean ± SD. Variables between groups were analyzed using the independent-samples Student's *t*-test or chi-squared tests and *p* <0.05 was considered statistically significant. ROC curve and area under the curve (AUC) analyses were performed to compare the percentage of MDSC among PBMC between groups. Odds ratios (ORs) and 95% CIs for a better IVF outcome associated with increased MDSC were estimated based on unconditional logistic regression.

## Results

### Subjects

**Figure 1:**
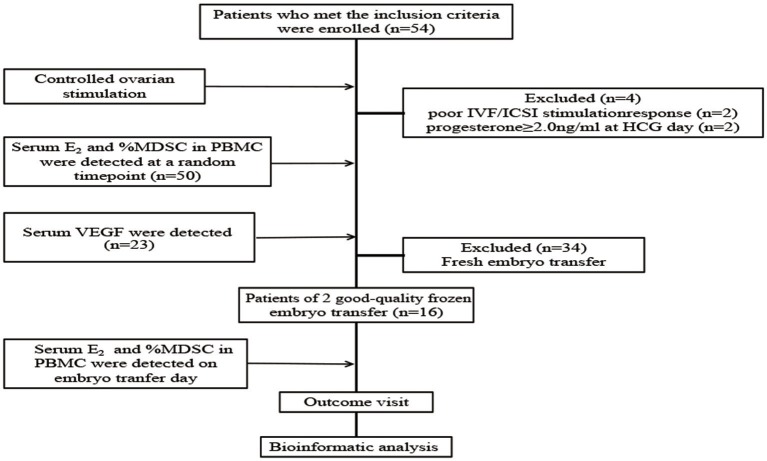
Flow chart of the study. Fifty-four patients were included, of which four patients were excluded from the analysis due to the exclusion criteria. Serum E_2_ level and percentage of MDSC among PBMC were detected in the remaining 50 patients at a random time-point during controlled ovarian stimulation, and serum VEGF level was detected (*n* = 23). Then, serum E_2_ level and percentage of MDSC in PBMC were detected on the embryo transfer day and pregnancy outcome visit were done in the patients of 2 good-quality frozen embryo transfer group (*n* = 16). Finally, we did bioinformatic analysis to predict the interaction between E_2_, VEGF and MDSC, and the probability of signal pathway.

Fifty-four patients who met the inclusion criteria were enrolled and treated with COS. Clinical characteristics are shown in Table 2. During COS, four patients were excluded from the analysis due to the following exclusion criteria: poor IVF/ICSI stimulation response (*n* = 2) and progesterone > 2.0 ng/ml at HCG day (*n* = 2). We then tested serum E_2_, percentage of MDSC on a random day during COS (*n* = 50), VEGF and S100A9 levels (*n* = 23). The crosstalk among these factors was also analyzed. To verify that MDSC ratios are predictive of IVF outcome independently, we tested E_2_ and MDSC levels in estrogen-primed FET patients on the day of FET. After transferring two good-quality embryos to each patient, the remaining 16 patients were divided into two groups according to MDSC level. No significant (*p* > 0.05) difference was found in these characteristics (Table 3), except for the percentage of MDSC among PBMC. Finally, bioinformatic analysis was conducted for further investigation (as outlined in a following section; Figure 1).

**Table 2:** Patients characteristics (*n* = 54).

**Characteristic**	**Mean**	**Median**	**SD**	**Range**
Age (years)	28.77	29.00	3.75	21–34
Duration of infertility (years)	3.64	3.00	2.72	0.5–12
BMI (kg/m^2^)	21.49	21.26	2.43	17.30–24.79
Basal level of FSH (mIU/ml)	6.63	6.53	1.48	3.06–9.62
Basal level of LH (mIU/ml)	4.70	4.81	1.55	1.59–8.48
Basal AFC	15.50	17	3.83	10–19
Total Gn (75IU)	21.16	19	6.32	10–42
Days of Gn	10.55	9	1.81	5–15
E_2_ on day of HCG (pg/ml)	3,006	2,304	2,845	9.6–12,175
P on day of HCG (pg/ml)	1.02	0.92	0.48	0.21–2.42
Em thickness on day of HCG (mm)	10.49	10	1.91	7–15
No. of retrieved oocytes	12.30	11	4.82	2–23
No. of fertilized oocytes	8.40	8	4.02	2–20
No. of good-quality embryos	5.44	5	2.73	2–12
No. of blastocyst	2.41	2	1.04	0–6

**Table 3:** Clinical and outcome of IVF patients.

**Characteristic**	**%MDSC in PBMC ≥ 8.22%**	**%MDSC in PBMC <8.22%**	***p*-value**
*n*	9	7	NA
Average age (Mean ± S.D.)	29.83 ± 2.75	31.00 ± 2.49	NS
Duration of infertility (years)	2.94 ± 0–68	3.57 ± 0.61	NS
BMI (kg/m^2^)	22.02 ± 0.68	21.66 ± 0.54	NS
Basal level of FSH (mIU/ml)	6.80 ± 0.52	6.78 ± 0.56	NS
Basal level of LH (mIU/ml)	5.67 ± 0.46	4.65 ± 0.84	NS
Basal AFC	15.33 ± 1.21	15.86 ± 1.70	NS
E_2_ on day of ET (pg/ml)	1502 ± 248.2	937.1 ± 262.8	NS
Em thickness on day of ET (mm)	10.44 ± 0.77	10.14 ± 0.94	NS
%MDSC in PBMC on day of ET (%)	14.05 ± 2.16	5.60 ± 0.78	0.0052
Pregnant	7	1	
Non-pregnant	2	6	
Pregnant rate (%)	77.78	14.29	

### E_2_, VEGF, S100A9, and MDSC Assays

**Figure 2:**
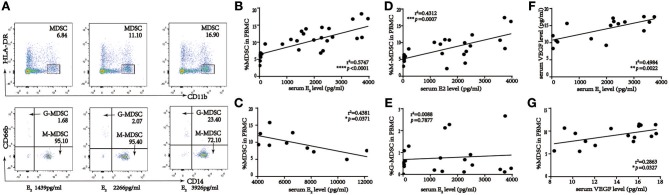
Percent of MDSC in the course of IVF patients, and statistical analysis of correlation among percentage of MDSC in PBMC, serum E_2_ and VEGF level in IVF patients. **(A)** Gating strategy of MDSC by flow cytometric analysis. HLA-DR^−/low^ and CD11b^+^ cells were first gated from live PBMC for MDSC. The expression of cell surface markers CD14^+^ and CD66b^+^ on this population was evaluated further for M-MDSC and G-MDSC subsequently. **(B)** Correlation between percentage of MDSC in PBMC and serum E_2_ level when serum E_2_ level < 4,000 pg/ml (*r*^2^ = 0.5747, ^****^p < 0.0001, *n* = 40). **(C)** Correlation between percentage of MDSC in PBMC and serum E_2_ level when serum E_2_ level more than 4,000 pg/ml (*r*^2^ = 0.4381, ^*^p = 0.0371, *n* = 10). **(D)** Correlation between percentage of M-MDSC in PBMC and serum E_2_ level when serum E_2_ level < 4,000 pg/ml (*r*^2^ = 0.4312, ^***^p = 0.0007, *n* = 40). **(E)** Correlation between percentage of G-MDSC in PBMC and serum E_2_ level when serum E_2_ level < 4,000 pg/ml (*r*^2^ = 0.0088, *p* = 0.7877, *n* = 40). **(F)** Correlation between serum VEGF and E_2_ level when serum E_2_ level < 4,000 pg/ml (*r*^2^ = 0.4984, ^**^p = 0.0022, *n* = 16). **(G)** Correlation between percentage of MDSC in PBMC and serum VEGF level when serum E_2_ level < 4,000 pg/ml (*r*^2^ = 0.2863, ^*^p = 0.0327, *n* = 16).

To investigate the correlation between MDSC and E_2_ levels during COS, we analyzed these parameters in blood and serum of women who underwent IVF treatment (*n* = 50). MDSC subsets were identified as CD11b^+^HLA-DR^low/−^ and CD66b^+^ for G-MDSC or CD14^+^ for M-MDSC populations. As shown in Figure 2A, the percentage of MDSC among PBMC was significantly elevated in IVF patients with elevated serum E_2_. Further, there was a positive correlation between serum E_2_ levels < 4,000 pg/ml and MDSC (*r*^2^ = 0.5747, ^****^p < 0.0001, *n* = 40; Figure 2B). However, an E_2_ concentration > 4,000 pg/ml was inversely proportional to MDSC, indicating that hyperphysiological E_2_ might be detrimental to MDSC recruitment (*r*^2^ = 0.4381, ^*^p = 0.0371, *n* = 10; Figure 2C). We further investigated the correlation between M-MDSC- or G-MDSC-dominant ratios and E_2_ (Figures 2D,E; *r*^2^ = 0.4312, ^***^p = 0.0007, and *r*^2^ = 0.0088, *p* = 0.7877, respectively, *n* = 40). Only M-MDSC showed a positive correlation, whereas there was no association with the G-MDSC group, when serum E_2_ levels were <4,000 pg/ml. Meanwhile, for E_2_ levels > 4,000 pg/ml, there was no obvious correlation between M-MDSC or G-MDSC and E_2_ (Supplemental Figures 1A,B).

As E_2_ plays an important role in regulating VEGF in normal uterine tissues (25) and VEGF is associated with MDSC recruitment, VEGF levels were measured in the E_2_ < 4,000 pg/ml (*n* = 16) and E_2_ > 4,000 pg/ml (*n* = 7) groups separately to understand associations among E_2_, VEGF, and the percentage of circulating MDSC in patients receiving IVF (Figures 2F,G, Supplemental Figures 1C,D). Serum E_2_ levels were correlated positively and negatively with VEGF in the <4,000 and >4,000 pg/ml groups, respectively, whereas VEGF levels and MDSC ratios were positively correlated for both groups. This indicated that E_2_ might regulate MDSC accumulation via VEGF.

The S100 calcium-binding proteins A8 and A9 (S100A8 and S100A9), also known as migration inhibitory factor-related proteins 8 (MRP8) and 14 (MRP14), are abundantly expressed in myeloid cells (26). The function of S100A9 in myeloid cells is still controversial (27), and thus, we detected its expression both *in vivo* and *in vitro* (Supplemental Figures 1E,F). In serum, ELISA results demonstrated that there was no obvious change of S100A9 levels in 2groups. *In vitro*, we treated PBMC with 0–100 μM 17β-estradiol for 6 days and used qRT-PCR to confirm these results, S100A9 expression was elevated under estradiol treatment, however there was no dose-dependent tendency.

### MDSC Suppress T Cell Responses in IVF Patients

The most important property of MDSC is their immunosuppressive activity (28). Therefore, we evaluated the effect of IVF-derived MDSC on T cell responses. MDSC were depleted from PBMC by flow cytometric sorting, after which the PBMC were stimulated with anti-CD3/CD28 antibodies. Compared to that with total PBMC, the results showed that the proliferation of both CD3^+^CD4^+^ and CD3^+^CD8^+^ T cells were enhanced significantly by MDSC depletion when serum E_2_ levels were more and less than 4,000 pg/ml, especially when more than 4,000 pg/ml (Supplemental Figure 2).

### 17β-Estradiol Augments MDSC *in vitro*

**Figure 3:**
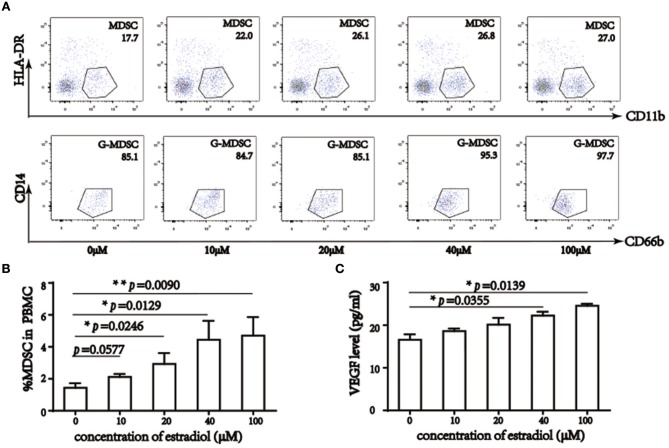
17β-estradiol induced MDSC augment and elevated VEGF level *in vitro*. **(A)** Flow cytometry analysis showed that percentage of MDSC in PBMC without lymphocyte, 17β-estradiol induced MDSC augment in a dose dependent manner. **(B)** Qualification of MDSC in total PBMC. **(C)** VEGF level in liquid supernatant under 17β-estradiol treatment. ^*^p <0.05, ^**^p < 0.01, ^***^p < 0.001.

To confirm the relationship between E_2_ and MDSC, we treated PBMC with 0–100 μM 17β-estradiol and cell subsets were detected by a flow cytometric assay. Previous studies have reported that lymphocytes varied with E_2_ treatment, with CD4^+^ T cells elevated and CD8^+^ T cells decreased (29–31). *In vitro* culture for 6 days led to alterations in PBMC subsets, and the morphology of M-MDSC was similar to that of monocytes, whereas G-MDSC was similar to neutrophils. Accordingly, based on *in vitro* analysis, we also demonstrated MDSC ratios in PBMC without lymphocytes (Figure 3A, Supplemental Figure 3) and MDSC percentage in PBMC (Figure 3B). The results showed that 17β-estradiol augmented MDSC numbers *in vitro*. Further, statistical analysis revealed that this increase was significant among the groups. At the same time, VEGF levels in the supernatant were enhanced obviously, especially when the concentration of 17β-estradiol was 40 μM (Figure 3C).

### MDSC as a Predicter of IVF Outcome

**Figure 4:**
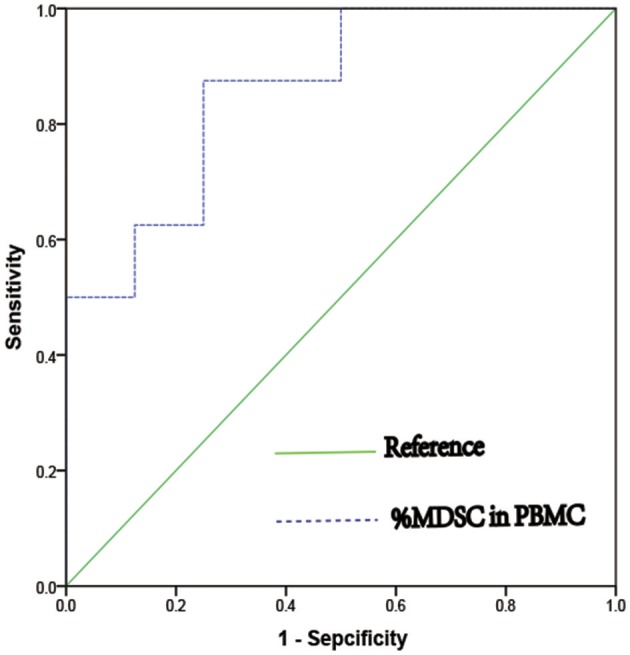
ROC curve of percentage of MDSC in PBMC as a predict marker of clinical pregnancy in women who undergoing IVF treatment (AUC = 0.859, 95%CI = 0.677 – 1.000, ^*^p = 0.016).

We next verified that MDSC ratios were predictive of IVF outcome independently; in the estrogen-primed FET patients, after transferring two good-quality embryos to each patient, we tested E_2_ and MDSC levels on the day of FET. ROC analysis (AUC = 0.859) comparing the percentage of MDSC in the peripheral blood between the clinically pregnant and non-clinically pregnant groups was performed (Figure 4). The optimal cut-off value proposed by ROC analysis for MDSC was 8.22%. Study patients were further divided into two groups based on this value as follows: MDSC ≥ 8.22% group (*n* = 9) and MDSC **<** 8.22% group (*n* = 7). There were no significant differences between the two groups regarding baseline characteristics; however, the clinical pregnancy rate in the MDSC ≥ 8.22% group was higher than that in the **<**8.22% group (77.78 vs. 14.29%, *p* < 0.05; Table 3). An increase in MDSC (≥8.22%) in the peripheral blood was also predictive of better IVF treatment outcome (95% CI: 0.677–1.00, ^*^p = 0.016), with a sensitivity of 87.5% and a specificity of 75.0%.

### Bioinformatic Analysis

**Figure 5:**
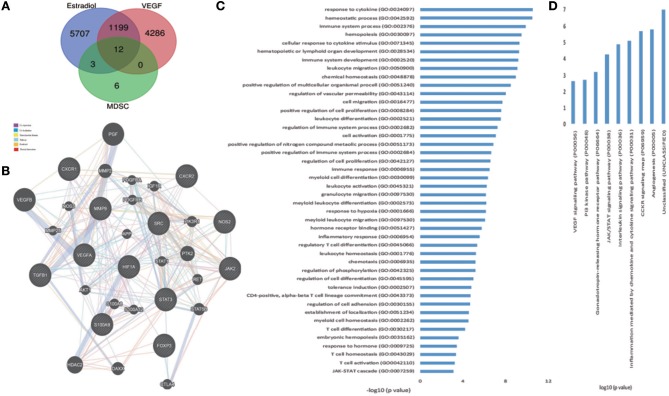
Hub genes of estradiol, VEGF, and MDSC, and bioinformatic analysis of the genes. **(A)** Venn diagrams of estradiol, VEGF, and functional genes in MDSC as hub genes. **(B)** Network of hub genes. Black protein nodes indicate target proteins, and different connecting colors represent different correlations. Functional association of targets was analyzed using GeneMANIA. Genes in black circles were submitted as query terms in searches. Gray circles indicate genes associated with query genes. **(C)** GO analysis of targets. The y-axis shows significantly enriched biological process categories of the targets, and the x-axis shows the enrichment scores of these terms (^*^p < 0.05). **(D)** Pathway analysis of targets. The y-axis shows significantly enriched pathway of the targets, and the x-axis shows the enrichment scores of these terms (^*^p < 0.05).

To further investigate and verify the mechanism associated with these interactions, we used bioinformatics software. CTD (The Comparative Toxicogenomics database) and the Coremine database can identify potential target proteins based on chemical–protein interaction analysis of small molecules. Both are powerful tools for computational target fishing. By utilizing CTD (http://ctdbase.org/) and COREMINE databases (http://www.coremine.com/medical/), we found 6,921 target genes related to E_2_ and 5,528 target genes associated with VEGF. Analysis revealed that *ARG1, IDO1*, and *NOS2* are considered functional genes for MDSC, whereas IL-6, IL-1A, IL-2, IL-17A, and FOXP3 are cytokines associated with T cells, which can be affected by MDSC. Further, results indicated that TGF1, HIF1A, JAK2, SRC, STAT3, COX2, GM-CSF, S100A8, and S100A9 might be involved in MDSC enrichment and accumulation, and that CXCR1 and CXCR2 could affect MDSC recruitment. Among these target genes, 12 common genes were identified based on a Venn diagram (Figure 5A, Table 4), in accordance with our previous experiment on VEGF and S100A9.

**Table 4:** Targets genes of E_2_, VEGF, and MDSC identified by CTD network.

**Rank**	**Gene symbol**	**Gene ID**	**Interaction count**
1	VEGFA	7422	62
2	TGFb1	7040	46
3	MMP9	4318	40
4	NOS2	4843	34
5	SRC	6714	15
6	STAT3	6774	12
7	JAK2	3717	8
8	S100A9	6280	8
9	FOXP3	50943	6
10	HIF1A	3091	5
11	CXCR2	3579	4
12	CXCR1	3577	4

GeneMANIA revealed interacting proteins that are functionally similar to the 12 genes; 39.26% had matching co-expression characteristics, 34.02% had similar co-localization, 15.95% shared identical protein domains, 7.78% were involved in the same pathways, and 1.46% displayed similar physical interactions (Figure 5B).

**Figure 6:**
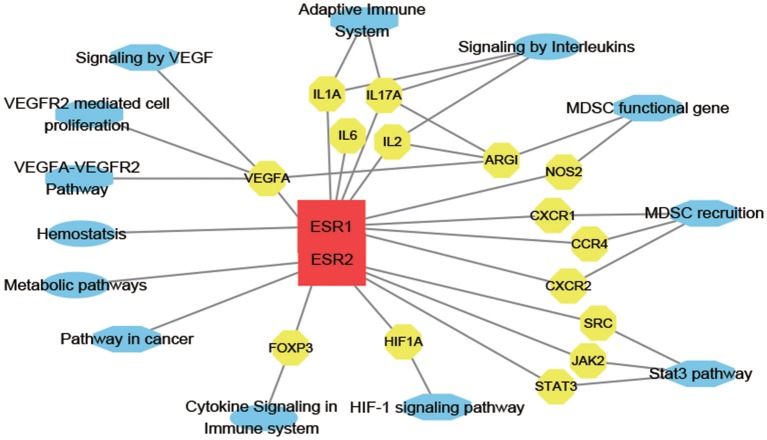
E_2_-target-pathway network. The interaction network has 30 nodes and 39 edges. The red square, yellow circle, and blue ellips, respectively, correspond to E_2_, target proteins and pathways.

To access probable signaling mechanisms, an analysis of interaction networks by DAVID showed that 93.44% of genes were enriched in biological processes such as immunological activities (GO:0002376), regulation of vascular permeability (GO:0043114), myeloid cell differentiation (GO:0030099), myeloid leukocyte migration (GO:0097529), and hormone receptor binding (GO:0051427) (Figure 5C). In addition, pathway analysis by DAVID showed that the targets were involved in angiogenesis (P00005), CCKR signaling map (P06959), JAK/STAT (P00038), and PI3K pathway (P00048) (Figure 5D). Further, based on target fishing and pathway analysis, an entire network was constructed using Cytoscape 3.0 (Figure 6).

## Discussion

Failed embryo implantation is one of the major limiting factors of successful IVF. Despite advances in techniques, there is an enduring problem of recurrent implantation failure (32). Successful implantation is dependent on the quality of embryos, receptivity of the endometrium, the influence of ovarian hormones, and the balance of the immune microenvironment (32, 33). The regulatory milieu of genes encoding processes like cell adhesion, cell differentiation, cell proliferation, angiogenesis, responses to stress, and signal transduction are considered critical for successful implantation (34). In addition, the interplay between anti- and pro-inflammatory and immune responses plays an important role in the acceptance of semi-allogenic embryos, as well as the establishment of a favorable environment for endometrium receptivity (35).

Circulating E_2_ changes in IVF patients undergoing COS correspond to the time of stimulation and the number of oocytes. Serum E_2_ levels influence not only the receptivity of the endomembrane during the implantation window, but also the development and activation of immune cells; in a sense, it can predict pregnancy outcome. Further, E_2_ signaling accelerates the progression of ovarian cancer by mobilizing MDSC and enhancing their intrinsic immunosuppressive activity, both *in vivo* and *in vitro* (36). However, it was not clear how E_2_ levels affect peripheral blood MDSC during IVF and if this could modulate IVF outcome.

Our results showed that with E_2_ < 4,000 pg/ml, with a lower incidence of ovarian hyperstimulation (OH), the percentage of MDSC correlated positively with both serum E_2_ and VEGF levels; *in vitro* experiments corroborated these results. Thus, we postulated that E_2_ might drive MDSC recruitment and augment VEGF levels. Growing evidence implicates E_2_ as a fundamental mediator of inflammation, which also affects VEGF and MDSC functions. During pregnancy, E_2_ influences helper CD4^+^ T cell differentiation favoring humoral Th_2_ over cell-mediated Th_1_ responses to protect the infant from maternal immune responses (37, 38). E_2_ also regulates VEGF in normal uterine tissues (25) and VEGFR1 and VEGFR2, expressed by MDSC, are crucial for MDSC recruitment and accumulation in a mouse model (39). Further, VEGFA knockdown in tumor cells results in decreased MDSC infiltration (19), indicating an indispensable role for VEFG in MDSC regulation. In addition, VEGF is a key regulator of angiogenesis and vascular function (18) at the time of receptivity and blastocyst implantation and for stromal decidualization (40). Moreover, it has been confirmed that in the tumor microenvironment, elevated E_2_ levels augment and enhance the immune-suppressive functions of MDSC via the IL-6–JAK2–STAT3 pathway (36). Recently, Verma et al. showed that in miscarriage patients, lower E_2_ and progesterone levels can result in STAT3 downregulation and lower MDSC proportion, which leads to a breakdown of maternal–infant tolerance (41). However, it is not clear how E_2_ levels affect MDSC and VEGF to alter the prognosis of IVF treatment.

It has been shown that in the absence of uterine VEGF, the uterus is not receptive to implantation (42). However, recent studies have shown a decrease in total VEGF in OH pregnancies (25, 43), indicating that OH disturbs VEGF and thus vascular permeability and decidualization (44). In line with previous studies, we found that at >4,000 pg/ml, E_2_ concentration was negatively related to VEGF and MDSC percentage in PBMC. In tumor environment, suppressive function of MDSC has been confirmed (28). Moreover, it has been verified that the suppressive function of MDSC can be enhance by E_2_ treatment, and as a dose-dependent manner (36), accordance with our study. Recently, several reports have provided evidence that S100A9 can exert anti-inflammatory effects by inducing the migration of MDSC in tumor-bearing individuals and autoimmune diseases (8, 45–47). Although a previous study demonstrated that S100A8 levels are elevated during the proliferative phase of the menstrual cycle, but decreased during the secretory phase, indicating that its expression is regulated by estrogen and that it can prevent overactivated inflammatory responses for successful implantation (48). However, our result showed no obvious correlation between S100A9 and E_2_ levels. This is possibly because VEGF has been described to cause an accumulation of MDSC, whereas S100A9 has only been shown to regulate their migration (27).

In healthy adults, no more than 5% MDSC is present among PBMC (49). However, a recent study observed the significant expansion of MDSC in the peripheral blood of pregnant women, which suppressed the T cells response (27, 50), indicating that MDSC might play a vital role in the maintenance of maternal–fetal tolerance. To verify whether the percentage of MDSC is predictive of IVF outcome, multiple logistic regression analysis was used to establish an independent predictive factor related to clinical pregnancy in this study. We found that MDSC levels comprised an independent predictive factor of clinical pregnancy, whereas the other tested parameters had no effect on IVF outcome for this cohort. Further statistical analysis was performed based on the percentage of MDSC above and below 8.22% to examine other variables, and demonstrated that high levels of MDSC in the peripheral blood are a better predictor of IVF outcome. One study demonstrated that G-MDSC are predictive of IVF clinical pregnancy (51), contradictory to the results of the present study. Based on our preliminary experiments (49, 52), blood sample treatment could play a crucial role in flow cytometric analysis, because there is still no effective marker to distinguish G-MDSC from neutrophils (53).

Bioinformatics analysis revealed 12 genes common among E_2_, VEGF, and MDSC that were mostly enriched in homeostatic and immune processes and correlated with myeloid cell differentiation and migration and also associated with hormone receptor binding and cell migration, adhesion, and localization-critical processes during embryo implantation. A drug-target association network was also generated by GO and pathway analysis. These results indicated that E_2_ has multiple pharmacological functions including immune regulation. Although further studies are necessary to confirm and assess these precise interactions, this study provides a systematic and visual overview of possible E_2_-mediated molecular signaling pathways involved in mitigating IVF failure.

Gene ontology, pathway analysis, and enrichment mapping of the endomembrane from COS patients and healthy controls previously revealed the significant dysfunction of immunological and inflammatory responses upon implantation failure (34). Moreover, the activation of a pro-inflammatory immune response, which is a requisite for a receptive endometrium, could be misdiagnosed leading to corticosteroid treatment to suppress the immune response, and resulting in IVF failure (54).

## Conclusion

In summary, we demonstrated that maternal–fetal interface immune tolerance is influenced by circulating E_2_ levels, which in turn regulates the MDSC population through VEGF and affects IVF outcomes. Further investigation of these important results will advance our understanding of the clinical application of MDSC as predictors of immune-related miscarriage and IVF failure.

## Data Availability

All datasets generated for this study are included in the manuscript and/or the Supplementary Files.

## Ethics Statement

The Ethics Review Boards of the First Hospital of Jilin University (No. 2018-069) and the Jilin Province People's Hospital (No.2018-Y-005) approved this research. Study participants and/or their legal guardians provided written informed consent.

## Author Contributions

CH and YZ: concepted the analysis and wrote the manuscript. BP: data collection and statistical analysis of the data. XL: involved in patient's treatment and review of manuscript. HY: funding acquisition, supervision of the study concept, and review of manuscript.

### Conflict of Interest Statement

The authors declare that the research was conducted in the absence of any commercial or financial relationships that could be construed as a potential conflict of interest.

## Supplementary Material

Supplemental Figure 1 Statistical analysis of correlation among percentage of MDSC in PBMC, E_2_, S100A9 and VEGF level. **(A)** Correlation between percentage of G-MDSC in PBMC and serum E_2_ level when serum E_2_ level more than 4,000 pg/ml (*n* = 10). **(B)** Correlation between percentage of M-MDSC in PBMC and serum E_2_ level when serum E_2_ level more than 4,000 pg/ml (*n* = 10). **(C)** Correlation between serum VEGF and E_2_ level when serum E_2_ level more than 4,000 pg/ml (*r*^2^ = 0.77, ^**^*p* = 0.0094, *n* = 7). **(D)** Correlation between percentage of MDSC in PBMC and serum VEGF level when serum E_2_ level more than 4,000 pg/ml (*r*^2^ = 0.5154, *p* = 0.0693, *n* = 7). **(E)** Serum S100A9 level in E_2_ level less and more than 4,000 pg/ml, respectively (*p* = 0.5821, *n* = 23). **(F)** Relative expression of S100A9 under different estradiol treatment.

Supplemental Figure 2 MDSC suppress T cell responses in IVF patients. **(A)** Proliferation of CD3^+^CD4^+^ T cells and CD3^+^CD8^+^ T cells in PBMC or PBMC without MDSC from peripheral blood of IVF patients. **(B)** Analysis of CD3^+^CD4^+^ T cells and CD3^+^CD8^+^ T cells proliferation in control, PBMC and PBMC without MDSC with different serum E_2_ level. **(C)** Statistical analysis of groups when serum E2 level less than 4,000 pg/ml. **(D)** Statistical analysis of groups when serum E2 level more than 4,000 pg/ml.

Supplemental Figure 3 17β-estradiol induced MDSC augment and elevated VEGF level *in vitro*. Flow cytometry analysis showed that percentage of MDSC in PBMC, 17β-estradiol induced MDSC augment in a dose dependent manner.
