# Supplementary figures and images for: Myeloid-Derived Suppressor Cells Are Regulated by Estradiol and Are a Predictive Marker for IVF Outcome
Source: Front Endocrinol (Lausanne). 2019 Jul 30;10:521. doi: 10.3389/fendo.2019.00521 (PMC6682648; doi:10.3389/fendo.2019.00521)

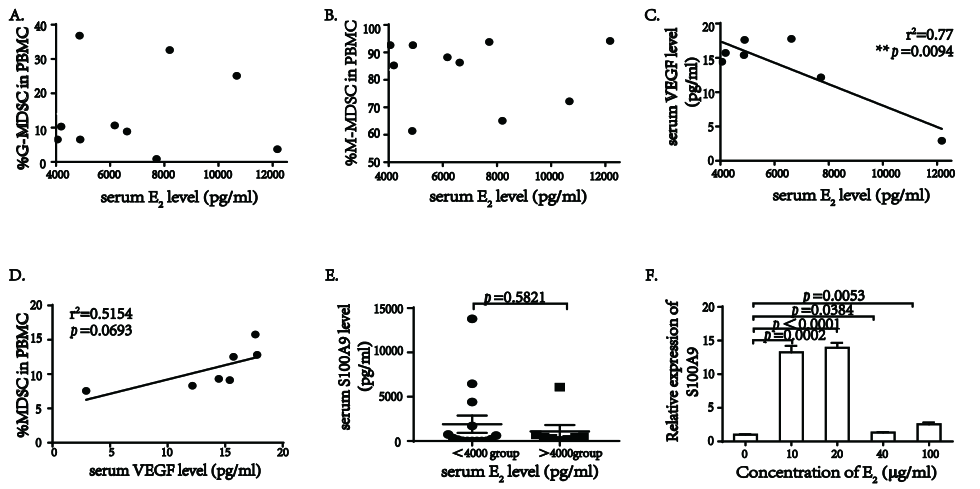

Supplement: Supplemental Figure 1 — Statistical analysis of correlation among percentage of MDSC in PBMC, E2, S100A9 and VEGF level. (A) Correlation between percentage of G-MDSC in PBMC and serum E2 level when serum E2 level more than 4,000 pg/ml (n = 10). (B) Correlation between percentage of M-MDSC in PBMC and serum E2 level when serum E2 level more than 4,000 pg/ml (n = 10). (C) Correlation between serum VEGF and E2 level when serum E2 level more than 4,000 pg/ml (r2 = 0.77, **p = 0.0094, n = 7). (D) Correlation between percentage of MDSC in PBMC and serum VEGF level when serum E2 level more than 4,000 pg/ml (r2 = 0.5154, p = 0.0693, n = 7). (E) Serum S100A9 level in E2 level less and more than 4,000 pg/ml, respectively (p = 0.5821, n = 23). (F) Relative expression of S100A9 under different estradiol treatment. [file Image_1.TIF]

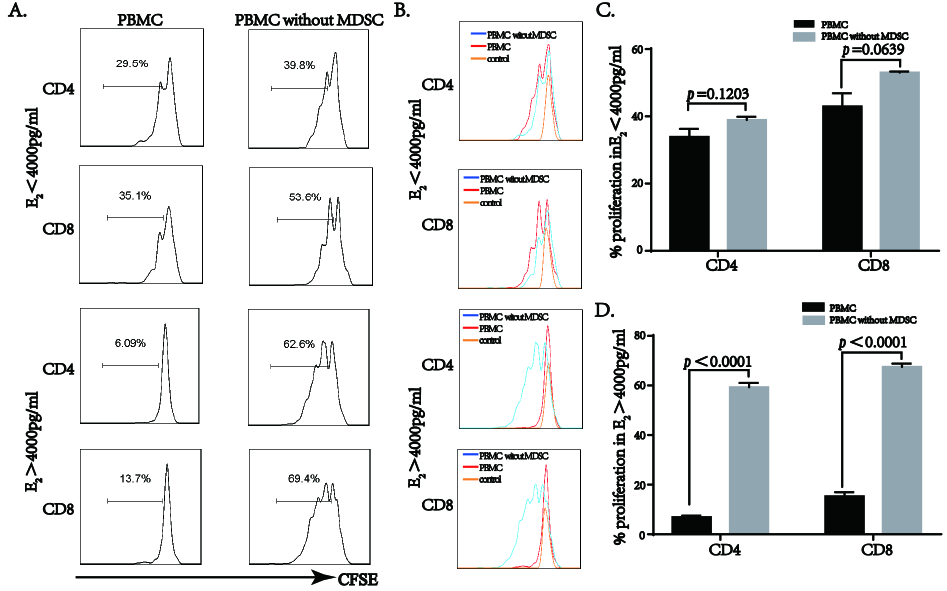

Supplement: Supplemental Figure 2 — MDSC suppress T cell responses in IVF patients. (A) Proliferation of CD3+CD4+ T cells and CD3+CD8+ T cells in PBMC or PBMC without MDSC from peripheral blood of IVF patients. (B) Analysis of CD3+CD4+ T cells and CD3+CD8+ T cells proliferation in control, PBMC and PBMC without MDSC with different serum E2 level. (C) Statistical analysis of groups when serum E2 level less than 4,000 pg/ml. (D) Statistical analysis of groups when serum E2 level more than 4,000 pg/ml. [file Image_2.TIF]

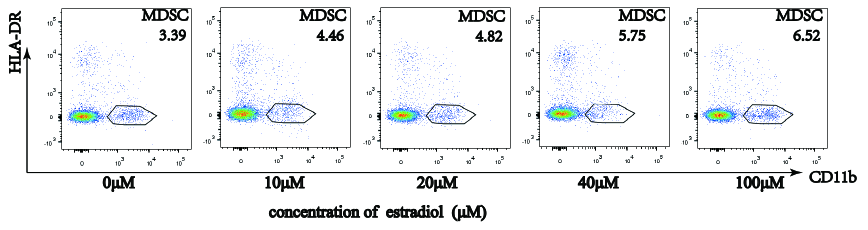

Supplement: Supplemental Figure 3 — 17β-estradiol induced MDSC augment and elevated VEGF level in vitro. Flow cytometry analysis showed that percentage of MDSC in PBMC, 17β-estradiol induced MDSC augment in a dose dependent manner. [file Image_3.TIF]
